# Occurrence of Hybrid *Escherichia coli* Strains Carrying Shiga Toxin and Heat-Stable Toxin in Livestock of Bangladesh

**DOI:** 10.3389/fpubh.2016.00287

**Published:** 2017-01-09

**Authors:** Fatema-Tuz Johura, Rozina Parveen, Atiqul Islam, Abdus Sadique, Md Niaz Rahim, Shirajum Monira, Anisur R. Khan, Sunjukta Ahsan, Makoto Ohnishi, Haruo Watanabe, Subhra Chakraborty, Christine M. George, Alejandro Cravioto, Armando Navarro, Badrul Hasan, Munirul Alam

**Affiliations:** ^1^International Center for Diarrheal Disease Research, Bangladesh (icddr,b), Dhaka, Bangladesh; ^2^Department of Microbiology, University of Dhaka, Dhaka, Bangladesh; ^3^National Institute of Infectious Diseases, Tokyo, Japan; ^4^Johns Hopkins Bloomberg School of Public Health, Baltimore, MD, USA; ^5^Department of Public Health, Faculty of Medicine, Universidad Nacional Autónoma de Mexico, Mexico City, Mexico; ^6^Infectious Disease, Department of Medical Sciences, Uppsala University, Uppsala, Sweden

**Keywords:** Shiga-toxin, enterotoxin, livestock, hybrid, multidrug resistant, PFGE

## Abstract

Shiga toxin-producing *Escherichia coli* (STEC) and enterotoxigenic *E. coli* (ETEC) are important causes of diarrhea in humans and animals worldwide. Although ruminant animals are the main source of STEC, diarrhea due to this pathotype is very low in Bangladesh where ETEC remains the predominant group associated with childhood diarrhea. In the present study, *E. coli* strains (*n* = 35) isolated from Bangladesh livestock (goats, sheep, and cattle) and poultry (chicken and ducks) were analyzed for the presence of major virulence factors, such as Shiga toxins (STX-1 and STX-2), heat-labile toxin, and heat-stable toxins (STa and STb). Multiplex polymerase chain reaction results revealed 23 (66%) *E. coli* strains to be virulent possessing either *sta* (*n* = 5), *stx* (*stx*1, *n* = 8; *stx*2, *n* = 2), or both (*n* = 8) genes in varying combinations. Thirty-four percent (8/23) of strains from livestock were hybrid type that carried both *stx* (either *stx*1 or *stx*2) and ETEC-specific enterotoxin gene *sta*. Serotyping results revealed that the ETEC strains belonged to five serotypes, namely O36:H5, O174:H−, O152:H8, O109:H51, and O8:H21, while the STEC-producing strains belonged to serotypes O76:H19 (*n* = 3), O43:H2 (*n* = 2), O87:H16 (*n* = 2), OR:H2 (*n* = 1), O110:H16 (*n* = 1), and O152:H8 (*n* = 1). The STEC–ETEC hybrid strains belonged to serotypes O76:H19 (*n* = 3), O43:H2 (*n* = 2), O87:H16, OR:H2, and O152:H8. Forty percent (2/5) of the ETEC and 20% (2/10) of the STEC strains were multidrug resistant with the highest drug resistance (50%) being found in the hybrid strains. Molecular fingerprinting determined by pulsed-field gel electrophoresis and cluster analyses by dendrogram revealed that, genetically, STEC–ETEC hybrid strains were highly heterogeneous. Multidrug-resistant *E. coli* STEC–ETEC hybrid strains in domesticated animals pose a public health threat for humans in Bangladesh.

## Introduction

*Escherichia coli* is commonly regarded as a non-pathogenic beneficial inhabitant of the gastrointestinal tract but several pathogenic strains acquired specific virulence factors that are accountable for a variety of intestinal and extra intestinal diseases, including diarrhea, acute inflammation, hemorrhagic colitis, urinary tract infections, septicemia, and neonatal meningitis (1–4). Based on virulence traits, major diarrheagenic *E. coli* pathogroups include shigatoxigenic *E. coli*, enterotoxigenic *E. coli* (ETEC), enteropathogenic *E. coli*, enteroinvasive *E. coli*, and enteroaggregative *E. coli* (EAEC) (3). Shiga toxin-producing *E. coli* (STEC) is a pathogen of significant public health concern and infection by this strain can result in a spectrum of outcomes ranging from asymptomatic carriage to severe diarrhea, as well as bloody diarrhea and hemolytic-uremic syndrome (HUS) (5). The disease causing ability of STEC is associated with the production of phage-encoded Shiga-like toxins (Stx), which are classified into two major families, Stx1 and Stx2 (encoded by *stx*1 and *stx*2 genes) on the basis of toxin neutralization assays and sequence analysis of *stx* genes. ETEC is the leading cause of diarrhea in travelers from industrialized countries and children living in low-income countries. ETEC bacteria are largely defined by the presence of the plasmid-encoded heat-labile (LT) and/or heat-stable (STa/STb) toxins (3).

Ruminants, especially cattle, sheep, and goat are the main source of STEC for humans and play an important role in the epidemiology of human infections (6). Meat contamination can facilitate transmission of pathogenic *E. coli* occurring through unhygienic slaughtering and poor food handling. Screening and characterization of animal STEC helps to identify the origin of human STEC infections. Despite the fact that over 400 STEC serotypes have been identified, only a subset of these have been correlated to human illness (7). Most outbreaks of hemorrhagic colitis and HUS have been associated with STEC O157 strains (5). There are outbreaks reported that are caused by non-O157 (8); infections caused by some non-O157 serotypes have also been frequently associated with severe illness in humans. In some geographic areas, STEC non-O157 strains are more commonly isolated than STEC O157 strains from persons with diarrhea or HUS (9). Antimicrobial resistance patterns may be an additional epidemiological marker for surveys of non-O157 SETC (10).

In human, ETEC strains are associated with one of the most frequent (sometimes fatal) causes of childhood diarrhea in the developing countries and an important causative agent of traveler’s diarrhea (3). ETEC is also a major cause of severe diarrheal disease in suckling and weanling animals (11). ETEC infections are transmitted through the fecal–oral route. Exposure to ETEC usually comes from contaminated food and drinking water (3). A close genetic relationship has been found between ETEC strains belonging to certain serotypes with diarrhea, which suggest that serotype analysis can be coupled with genetic typing to study strain clustering in epidemiologic and pathogenic studies. To assess the potential public health risk posed by ETEC and STEC, the detection of virulence factors, such as heat-stable toxin a (*STa*), heat-stable toxin b (*STb*), heat-labile toxin (*LT*), *stx1*, and *stx2*, is recommended (12–16).

Shigatoxigenic *E. coli* and other diarrheagenic *E. coli* are capable of acquiring virulence and related genes *via* horizontal gene transfer leading to the development of pathogroups different from the pre-existing ones (17). Such divergent pathogroups are often reported using different terminology such as “hybrid” (18), “blended virulence profiles,” and “virulence combination” (19). Several studies have reported coexisting STEC- and ETEC-associated virulence genes in *E. coli* strains of human, animal, and environmental origins (17, 20–25). The notorious sprout-borne outbreak strain O104:H4 in Germany possessed EAEC- and STEC-associated virulence genes (18) pointing to the possibility of extremely pathogenic strains emerging over a short time period (26).

In Bangladesh, the prevalence of STEC in hospitalized patients with diarrhea is very low (0.5%) (27), but it was somewhat higher among community cases with diarrhea (1.9%) (27). In Bangladesh, the predominant group of *E. coli* associated with childhood diarrhea is ETEC, which accounts for approximately 20% of all diarrheal cases (28). Animals seem to be the potential carrier for pathogenic *E. coli* but there is little data available concerning animal reservoirs, epidemiology, human pathogenicity, and drug resistance profiles of STEC and ETEC in Bangladesh. In the present study, we report the existence of multidrug-resistant *E. coli* strains belonging to STEC, ETEC, and a newly emerged STEC–ETEC hybrid in domesticated animals, which pose a potential public health threat for humans in Bangladesh.

## Materials and Methods

### Sample Collection, Isolation, and Identification of *E. coli*

Rectal (livestock) and cloacal (bird) samples were collected from a total of 35 different livestock [goat (*n* = 19), sheep (*n* = 9), cattle (*n* = 2)] and poultry [chicken (*n* = 3) and duck (*n* = 2)] between June and August 2007 from the district of Mymensingh, Bangladesh. Sterile cotton swabs were used for the collection of rectal and cloacal samples. After collection, the cotton swabs were dipped into Carry-Blair media and stored in a cool box (4°C). Immediately after sampling, all samples were transported to the laboratory for analysis. All samples were streaked onto MacConkey agar (Difco), and the plates were incubated overnight at 37°C. Bright pink lactose-fermenting colonies were selected as presumptive *E. coli*. These colonies were again grown on eosin methylene blue agar to examine for the production green colonies with a metallic sheen. One presumptive colony per sample was identified by biochemical tests, as described before (29).

### Polymerase Chain Reaction (PCR) Amplification of Virulence Genes

DNA was extracted from isolated colonies suggestive of *E. coli*, as described previously (30). The samples were then subjected to molecular analysis of ETEC (*sta, stb, elt*) and STEC (*stx1, stx2*) virulence genes using multiplex PCR. The PCR assay conditions are described in Table 1. Each amplification was conducted in a volume of 20 µl containing the following reagents: 1 µl template DNA, 0.5 µl dNTP (Invitrogen, Carlsbad, CA, USA) solution (10 mmol 1^−1^), 1.0 µl of each primer at 5 pmol l^−1^, 0.6 µl Taq DNA polymerase solution (Invitrogen) (1.5 U μl^−1^), 2.0 µl PCR reaction buffer solution (10×) with 0.8 µl MgCl_2_ (50 mmol l^−1^), and presterilized ultrapure water (Milli-Q) to 20 µl. This mixture was processed in a thermocycler at 94°C for 4 min (denaturation), followed by 30 cycles at 94°C for 1 min (denaturation), annealing temperature (specific for each primer) for 1 min (binding), and at 72°C for 1.5 min (extension). Complete extension of the Taq DNA polymerase was performed at 72°C for 7 min. An aliquot of this reaction containing only water without DNA was used as a negative control. Two multiplex PCR tests were carried out; one for the primer set LT, STa, and STb and the other for the primer set *Stx1* and *Stx2*.

**Table 1:** **Primer sequences for *STa, STb, LT, Stx1*, and *Stx2* genes, binding temperature, size of the amplification product, and reference of each primer used**.

Primers	Sequence	Annealing temperature (°C)	Size of the amplification product (bp)	Reference
*STa*	TCC CCT CTT TTA GTC AGT CAA CTG	57	163	Ngeleka et al. (12)
	GCA CAG GCA GGA TTA CAA CAA AGT			
*STb*	GCA ATA AGG TTG AGG TGA T		368	Lortie et al. (13)
	GCC TGC AGT GAG AAA TGG AC			
*LT*	TTA CGG CGT TAC TAT CCT CTC TA		275	Furrer et al. (14)
	GGT CTC GGT CAG ATA TGT GAT TC			
*Stx1*	AGA GCG ATG TTA CGG TTT G	55	388	Jackson et al. (15)
	TTG CCC CCA GAG TGG ATG			
*Stx2*	TGG GTT TTT CTT CGG TAT C		807	Jackson et al. (16)
	GAC ATT CTG GTT GAC TCT CTT			

### Serotyping

The rabbit antisera against O1 to O187strains were prepared in rabbits (SERUNAM) according to procedures described by Ewing (31). The *E. coli* O1 to O172 strains were obtained from the Laboratory of Gastrointestinal Pathogens, Department of Gastrointestinal, Emerging and Zoonotic Infections, Centre for Infections, Health Protection Agency, London, UK, and the *E. coli* O173 to O186 strains (32) were obtained from the Statens Serum Institute, Copenhagen.

The strains were serotyped by agglutination assay (33) using 96-well micro titer plates and rabbit serum (SERUNAM) obtained against 187 somatic antigens and 53 flagellar antigens for *E. coli*.

### Antibiotic Susceptibility

Susceptibility to antibiotics was performed by disk diffusion, as described by Bauer et al. (34) and the Clinical and Laboratory Standards Institute (35) (CLSI), using commercial antibiotic discs. Thirteen antibiotics (Oxoid, UK) were employed: erythromycin (E, 15 µg); gentamicin (CN, 10 µg); trimethoprim/sulfamethoxazole (SXT, 30 µg), tetracycline (TE, 30 µg), ampicillin (AMP, 30 µg), streptomycin (S, 10 µg), azithromycin (AZM, 15 µg), nalidixic acid (NA, 30 µg), ciprofloxacin (CIP, 5 µg), ceftriaxone (CRO, 30 µg), cefixime (CFM, 5 µg), mecillinam (MEL, 25 µg), and cephalothin (KF, 30 µg). Characterizations of the resistance or susceptibility profiles of the isolates were determined by measuring the inhibitory zone and comparing it with an established interpretative chart to determine sensitivity to each antibiotic.

### Pulsed-Field Gel Electrophoresis (PFGE)

Whole agarose-embedded genomic DNA from the *E. coli* isolates was prepared. PFGE was carried out using a contour-clamped homogeneous electrical field (CHEF-DRII) apparatus (Bio-Rad), according to procedures described previously (36). Genomic DNA of the test strains and *Salmonella enterica* serovar Braenderup was digested using *Xba*I, with fragments employed as molecular size markers. Restriction fragments were separated in 1% pulsed-field-certified agarose in 0.5× TBE (Tris–borate–EDTA) buffer. Post-electrophoresis gel treatment included both gel-staining and de-staining. The DNA was visualized using a UV transilluminator, and images were digitized *via* a one-dimensional gel documentation system (Bio-Rad).

### PFGE Analysis

The fingerprint pattern in the gel was analyzed using a computer software package, Bionumeric (Applied Maths, Belgium, version 3.1). After background subtraction and gel normalization, the fingerprint patterns were typed according to banding similarity and dissimilarity, using the Dice similarity coefficient and unweighted-pair group method employing average linkage (UPGMA) clustering, as recommended by the manufacturer. The results were graphically represented as dendrograms.

## Results

### Phenotypic Characteristics of *E. coli*

All the *E. coli* strains (*n* = 35) included in this study produced bright pink colonies on MacConkey and green colonies with metallic sheen on EMB agar plates. All strains gave biochemical reactions typical of *E. coli*.

### Virulence Gene Profile

Among the 35 *E. coli* strains isolated from livestock and poultry in the present study, 23 (66%) (goats: 17, sheep: 4, chicken: 1, and ducks: 1) carried virulence genes, such as, *sta* and *stx*, either *stx1* or *stx2*, but did not carry *stb* or *elt* genes, as confirmed by PCR (Table 2). Of the 23 strains carrying toxin genes, 5 (22%) had *sta*, and 10 (44%) had *stx* genes. Of the 10 *E. coli* carrying *stx* gene, 8 (80%) harbored only *stx*1, and 2 (20%) had only *stx2*. Eight of the 23 (34%) toxigenic strains were hybrid of which 7 (88%) possessed both *sta* and *stx1*, while the remaining strain carried both *sta* and *stx2* (Table 2). None of the strains tested presented *elt* or *stb*.

**Table 2:** **Serotypes, virulence gene, and drug resistance pattern of *E. coli* isolated from livestock samples in 2007, Bangladesh**.

No. of isolates	Virulence genes					Serotype	Resistance pattern	Source
	*Sta*	*Stb*	*Lt*	*Stx1*	*Stx2*			
1	+	−	−	−	−	O36:H5	E^R^, AZM^R^	Goat
1	+	−	−	+	−	O43:H2	E^R^	Goat
2	−	−	−	+	−	O43:H2	E^R^	Goat
1	+	−	−	+	−	O43:H2	E^R^, AZM^R^, S^R^	Goat
1	−	−	−	+	−	O76:H19	E^R^	Goat
1	+	−	−	+	−	O76:H19	E^R^, KF^R^	Goat
1	+	−	−	+	−	O76:H19	Sensitive to all antibiotics	Goat
1	−	−	−	+	−	O76:H19	E^R^	Goat
1	+	−	−	+	−	O76:H19	E^R^, AZM^R^, S^R^	Goat
1	−	−	−	+	−	O76:H19	E^R^, AZM^R^, S^R^, KF^R^	Goat
1	−	−	−	−	+	O87:H16	E^R^	Goat
1	+	−	−	−	+	O87:H16	E^R^, SXT^R^, AZM^R^	Goat
1	−	−	−	−	+	O87:H16	E^R^, SXT^R^, AZM^R^, S^R^	Goat
1	+	−	−	−	−	O174:H−	E^R^, SXT^R^, AZM^R^, KF^R^	Goat
1	−	−	−	+	−	OR:H2	E^R^	Goat
1	+	−	−	+	−	OR:H2	E^R^, CIP^R^, SXT^R^, NA^R^, AZM^R^, TE^R^, KF^R^	Goat
2	−	−	−	−	−	ND	ND	Goat
1	−	−	−	+	−	O110:H16	E^R^	Sheep
1	+	−	−	−	−	O152:H8	E^R^, S^R^	Sheep
1	−	−	−	+	−	O152:H8	Sensitive to all antibiotics	Sheep
1	+	−	−	+	−	O152:H8	Sensitive to all antibiotics	Sheep
5	−	−	−	−	−	ND	ND	Sheep
2	−	−	−	−	−	ND	ND	Cattle
1	+	−	−	−	−	O109:H51	E^R^, CIP^R^, SXT^R^, NA^R^, AZM^R^	Chicken
2	−	−	−	−	−	ND	ND	Chicken
1	+	−	−	−	−	O8:H21	E^R^, AZM^R^	Duck
1	−	−	−	−	−	ND	ND	Duck

*E, erythromycin; AZM, azithromycin; S, streptomycin; KF, cephalothin; SXT, trimethoprim/sulfamethoxazole; CIP, ciprofloxacin; NA, nalidixic acid; TE, tetracycline; R, resistant; ND, not done*.

### Serotyping of *E. coli*

**Figure 1:**
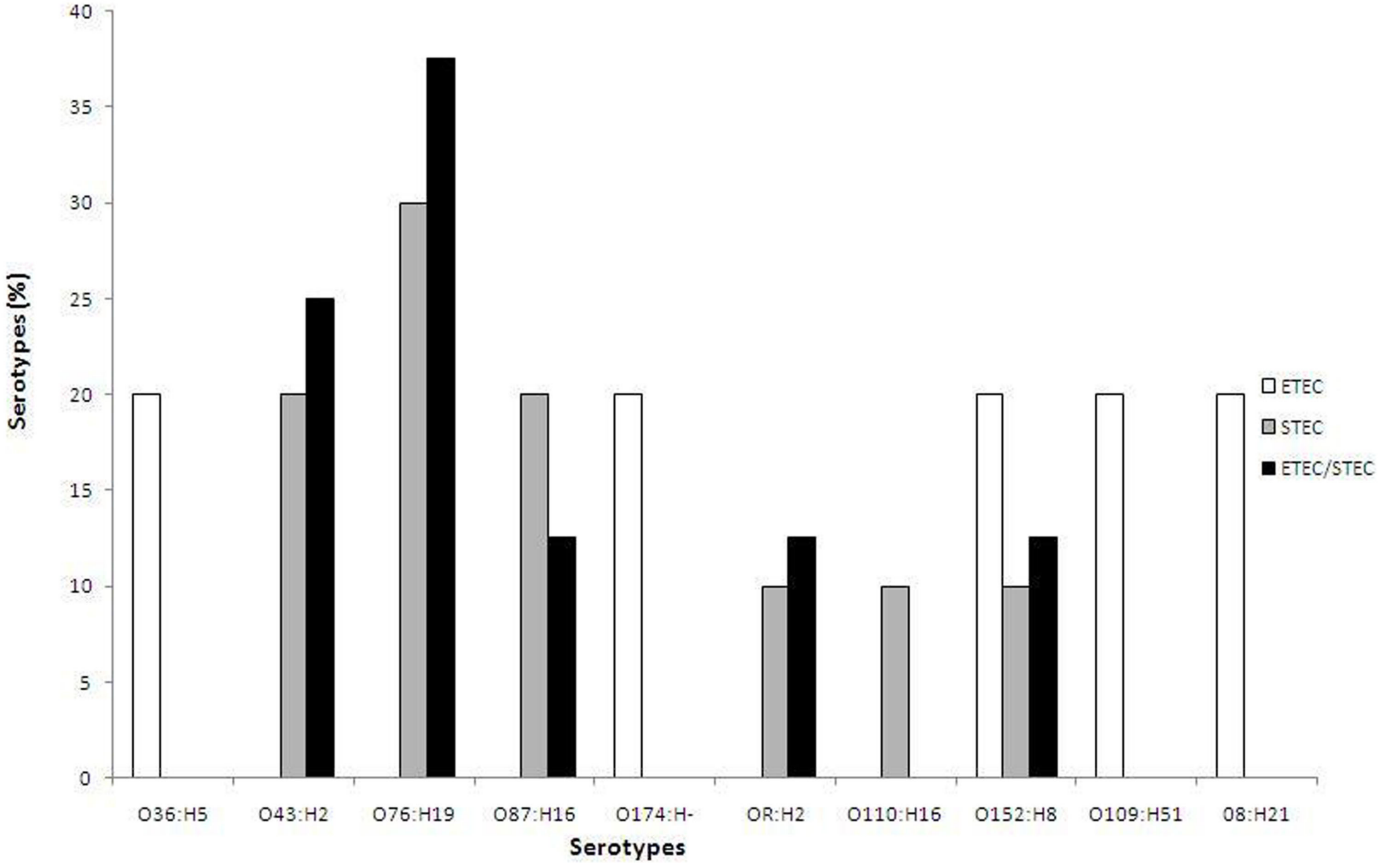
**Percentage of different serotypes of three *E. coli* pathogroups [enterotoxigenic *E. coli* (ETEC), STEC, and ETEC–STEC hybrid] from livestock animals (goat and sheep) and poultry (chicken and duck) in Bangladesh**.

Based on serotyping, the 23 toxigenic *E. coli* strains belonged to 10 different O serogroups [O8 (*n* = 1), O36 (*n* = 1), O43 (*n* = 4), O76 (*n* = 6), O87 (*n* = 3), O109 (*n* = 1), O110 (*n* = 1), O152 (*n* = 3), O174 (*n* = 1), and OR (*n* = 2)] and 8 different H seogroups [H2 (*n* = 6), H5 (*n* = 1), H8 (*n* = 3), H16 (*n* = 4), H19 (*n* = 6), H21 (*n* = 1), H51 (*n* = 1), and H− (*n* = 1)]. *E. coli* strains carrying virulence genes belonged to 10 different serotypes: O36:H5 (*n* = 1), O43:H2 (*n* = 4), O76:H19 (*n* = 6), O87:H16 (*n* = 3), O174: H− (*n* = 1), OR: H2 (*n* = 2), O110:H16 (*n* = 1), O152:H8 (*n* = 3), O109:H51 (*n* = 1), and O8:H21 (*n* = 1). None of the tested strains belonged to the EHEC serogroup, as they did not agglutinate with the antisera specific for O157 (Table 2). The ETEC strains (*n* = 5) belonged to five different serotypes: O36:H5, O174: H−, O152:H8, O109:H51, and O8:H21, while the STEC strains (*n* = 10) belonged to six different serotypes: O76:H19 (30%), O43:H2 (20%), O87:H16 (20%), OR: H2 (10%), O110:H16 (10%), and O152:H8 (10%) (Figure 1). In the order of prevalence of the STEC–ETEC hybrid strains (*n* = 8), O76:H19 was the most prevalent (37.5%) serotype, followed by O43:H2 (25%), O87:H16 (12.5%), OR: H2 (12.5%), and O152:H8 (12.5%) (Figure 1).

### Antibiotic Assay

Antibiotic susceptibility assay was performed for the 23 toxigenic strains only, and the results showed 87% of the strains to be resistant to erythromycin, 43% to azithromycin, 22% to streptomycin, 22% to trimethoprim/sulfamethoxazole, 17% to cephalothin, 9% to ciprofloxacin, 9% to nalidixic acid, and 4% to tetracycline (Table 2). All *E. coli* strains were sensitive to gentamicin, ampicillin, ceftriaxone, cefixime, and mecillinam. Results also revealed 40% (2/5) of the ETEC and 20% (2/10) of the STEC strains to be multidrug resistant (MDR) showing resistance to erythromycin, trimethoprim/sulfamethoxazole, azithromycin, cephalothin, ciprofloxacin, and nalidixic acid. The highest MDR was found in the hybrid strains, accounting for 50% (4/8) (Table 2), although the strains varied in their patterns of response to the different drugs tested.

Overall drug response results revealed 11 different resistance patterns among the three pathogroups of *E. coli* (ETEC, STEC, and hybrids of STEC–ETEC). Resistance patterns I (E^R^, S^R^), II (E^R^, AZM^R^), III (E^R^, SXT^R^, AZM^R^, KF^R^), and IV (E^R^, CIP^R^, SXT^R^, NA^R^, AZM^R^) were found in ETEC; resistance patterns V (E^R^), VI (E^R^, AZM^R^, S^R^, KF^R^), and VII (E^R^, SXT^R^, AZM^R^, S^R^) in STEC; and pattern V (E^R^), VIII (E^R^, KF^R^), IX (E^R^, AZM^R^, S^R^), X (E^R^, SXT^R^, AZM^R^), and XI (E^R^, CIP^R^, SXT^R^, NA^R^, AZM^R^, TE^R^, KF^R^) among the STEC–ETEC hybrid pathotypes (Table 3).

**Table 3:** **Antibiotic resistance profiles of the toxigenic *E. coli* (*n* = 23) isolates**.

Pathotype/virulence gene	Type	Antibiotic resistance profile	No. of strains
Enterotoxigenic *Escherichia coli* (ETEC) (*sta*^+^, *n* = 5)	I	E^R^, S^R^	1
	II	E^R^, AZM^R^	2
	III	E^R^, SXT^R^, AZM^R^, KF^R^	1
	IV	E^R^, CIP^R^, SXT^R^, NA^R^, AZM^R^	1
STEC (*stx*1^+^ or *stx*2^+^, *n* = 10)	V	E^R^	7
	VI	E^R^, AZM^R^, S^R^, KF^R^	1
	VII	E^R^, SXT^R^, AZM^R^, S^R^	1
		Sensitive to all antibiotics	1
STEC–ETEC hybrid (*sta*^+^ and *stx*1^+^ or *stx*2^+^, *n* = 8)	V	E^R^	1
	VIII	E^R^, KF^R^	1
	IX	E^R^, AZM^R^, S^R^	2
	X	E^R^, SXT^R^, AZM^R^	1
	XI	E^R^, CIP^R^, SXT^R^, NA^R^, AZM^R^, TE^R^, KF^R^	1
		Sensitive to all antibiotics	2

*E, erythromycin; AZM, azithromycin; S, streptomycin; KF, cephalothin; SXT, trimethoprim/sulfamethoxazole; CIP, ciprofloxacin; NA, nalidixic acid; TE, tetracycline; R, resistant*.

### PFGE and Cluster Analysis

**Figure 2:**
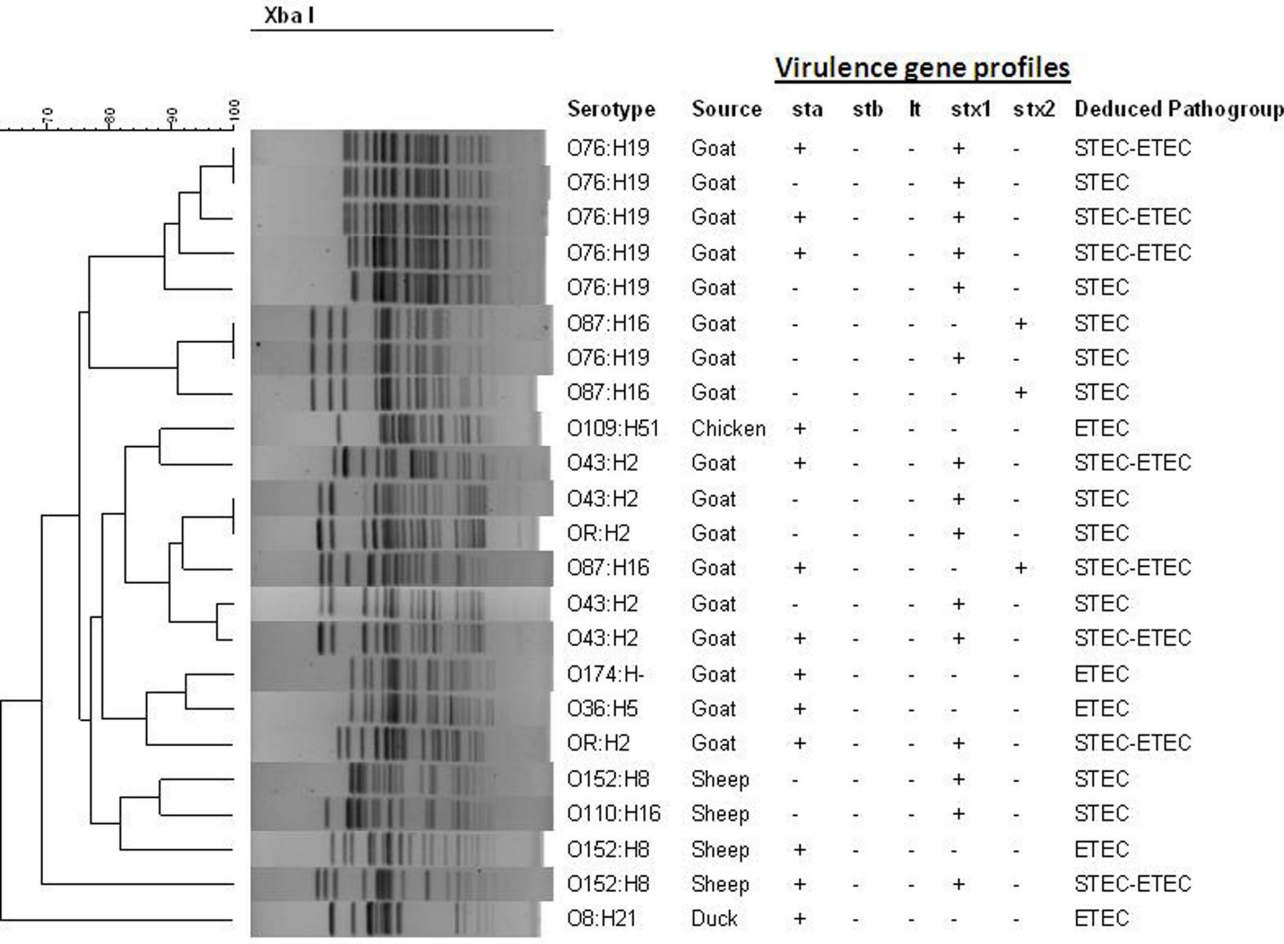
**Pulsed-field gel electrophoresis (PFGE) patterns of *Xba*I-digested genomic DNA of potentially pathogenic *E. coli* strains (*n* = 23) isolated from different livestock’s animal and poultry of Bangladesh in 2007**. Serotypes, source, and virulence gene content are indicated. The dendrogram was prepared by the BioNeumeric software (Applied Maths) using dice similarity coefficient and unweighted-pair group method employing average linkage of the PFGE images of the *E. coli* strains. The scale bar at the top (left) indicates similarity coefficient (%).

The *Xba*I-digested genomic DNAs of the *E. coli* strains carrying virulence genes from animal and poultry were subjected to PFGE to determine genetic relatedness and clonal origin. The number of fragments generated by restriction digestion with *Xba*I varied between 14 and 19, and the molecular size of the fragments ranged from 30 to 600 kb. Diverse PFGE patterns obtained from the 23 toxigenic *E. coli* strains (Figure 2) belonged to multiple small sub-clusters (Figure 2) when dendrograms were prepared with PFGE images by the BioNeumeric software (Applied Maths) using dice similarity coefficient and UPGMA. Although the *E. coli* strains exhibited high genetic heterogeneity in the PFGE patterns, four hybrid strains belonging to serotype O76:H19 formed a small tight cluster, suggesting that genetically, they are closely related.

## Discussion

### Occurrence of Hybrid of STEC and ETEC Strains

Enterotoxigenic *E. coli* and Shiga toxin-producing *E. coli* (STEC) are notorious pathogens associated with severe diarrhea and HUS in humans. Although *E. coli* constitutes an important component of normal gut bacterial flora, many studies have shown the non-toxigenic strains to harbor a number of different virulence factors and to be diarrheagenic (17, 18, 37). Here, we present data showing the prevalence of *E. coli* strains carrying an ETEC-specific gene, *sta*, and STEC-specific gene, *stx*, which included either *stx1* or *stx2* in the gut of livestock and poultry in Bangladesh. This study also presents data showing for the first time that a significant proportion (8/23) of the toxigenic *E. coli* strains from livestock were MDR and hybrid type carrying both *stx* (either *stx*1 or *stx*2) and ETEC-specific enterotoxin gene, *sta*. The presence of such a high percentage of MDR hybrid type *E. coli* strains in livestock poses a potential health threat to people of Bangladesh.

### Virulence Gene Profiling of *E. coli* Strains

Results obtained in this study confirmed that a very high proportion of the *E. coli* strains (66%) deriving from different livestock are toxigenic, carrying either *sta, stx*, or both in varying combinations. What is most important to note is that a significant proportion of *E. coli* in livestock was confirmed to be ETEC, including 22% carrying the *sta* gene only, 44% STEC (harboring only *stx* gene; either *stx*1 or *stx*2), and 35% hybrid comprising both *est* and *stx*. ETEC is an important cause of diarrhea in humans and animals in the low-income countries and is the main cause of diarrhea in travelers to low-income countries (1). One study carried out in Spain reported a high incidence of ETEC (30%) in piglets with diarrhea (38). A study carried out in Bangladesh revealed 61% of the *E. coli* strains isolated from the aquatic environment carried the *est* gene (39). Zoonotic and environmental transmission may be the reason ETEC is the predominant group of *E. coli* associated with childhood diarrhea in Bangladesh (28). The occurrence of ETEC in farm animals has always been of great public health importance as animals excreting pathogenic bacteria can contaminate water bodies in and around the area they are reared and in many ways can be related to human diseases. This may be one reason why ETEC is largely a pathogen of low-income countries where access to safe drinking water is often low.

STEC strains can cause bloody diarrhea known as haemorrhagic colitis, non-bloody diarrhea, and HUS (1). The data presented in this study provide evidence that livestock is an important reservoir for STEC carrying either *stx1* or *stx2*. A study carried out in Bangladesh reported 37.9% of buffalo, 20.1% of cows, and 10.0% of goats were positive for STEC (40). Our data appear to concur with 44% (*n* = 10) of livestock *E. coli* isolated mostly from goats (80%, 8/10 or 35%, 8/23) revealed STEC, either stx1 or stx2. Our results also appear to agree with those from a study carried out in Vietnam that showed STEC to account for 27% of *E. coli* isolated from buffalos, 23% from cattle, and 38.5% from goats (41). Although water plays an important role in the transmission of pathogenic bacteria, a study carried out in Bangladesh reported the presence of *stx* only in 1.25% of the *E. coli* strains isolated from natural aquatic environments (39). STEC-associated diarrhea is also very low compared to that of other enteric pathogens, including *Vibrio cholerae* and ETEC in Bangladesh (27). This low prevalence of STEC as the etiology of diarrhea could be due to STEC-specific antibodies in the human population, as this pathotype is present in a high proportion of *E. coli* occurring in domesticated livestock, such as goats and sheep, although it was not found in poultry, and only rarely in surface water samples (39). The low prevalence in natural surface water could be related to the incompetence of the STEC strains to survive beyond their livestock reservoirs (cattle, sheep, and goats) (6). Despite the low prevalence of STEC infections in Bangladesh (27), the virulence potential of these livestock strains to emerge as a novel STEC pathogen cannot be ruled out.

In the present study, 34% of the toxigenic livestock *E. coli* strains were hybrid as those carried toxin genes present in two different pathotypes; *stx* and *est*. Notably, majority of these hybrid strains (7/8) carried both *sta* and *stx 1*, while one strain carried both *sta* and *stx2* genes. This finding is not unique to our study as a number of past studies have shown hybrid strains carrying virulence marker genes of two different *E. coli* pathotypes (17), nonetheless, in a significantly lower frequency (2%) than that we observed in Bangladesh. Clinically linked STEC–ETEC hybrid *E. coli* strains from Brazil, Denmark, France, Germany, Great Britain, Mexico, and the United States were shown to harbor virulence markers for two or three different pathotypes (17). In a study in Germany, 0.6% of the human STEC isolates possessed *stx2g* and *estIa* (37). Serotype O100:H of a STEC–ETEC hybrid strain carrying *stx2* and *estIa* genes was isolated from contaminated drinking water during an outbreak in Finland (22). A later study reported a STEC strain carrying *stx1a* and *estIa* from cattle in Burkina Faso (42). Although no data exist on the virulence potential of STEC–ETEC hybrid strains isolated from livestock in Bangladesh, the widespread distribution and clinical relevance worldwide might indicate their virulence potential.

### Serodiversity

In the present study, serotyping results revealed that the ETEC strains belonged to five different serotypes: O36:H5, O174:H−, O152:H8, O109:H51, and O8:H21. No sero-specificity was observed for these ETEC strains as there was no relationship between the serotypes and the type of toxin genes (*st* or *lt*) they possessed. Likewise, the STEC strains belonged to six different serotypes (O76:H19, O43:H2, O87:H16, OR: H2, O110:H16, and O152:H8); STEC O157:H7 serotype was not present among the tested strains. Moreover, the STEC serotypes did not belong to any of the serotypes of the big six groups (O26, O45, O103, O111, O121, and O145) in the present study (43). STEC non-O157 isolates have been shown to be important pathogens despite having been underreported because in many laboratories, the facilities to isolate, identify, and characterize this pathotype do not exist (40). One report suggested that some non-O157:H7 STEC strains cause human illness and likely account for 20–50% of STEC infections (an estimated 37,000 cases) annually in the United States (44). In our study, we have identified eight *E. coli* strains that were hybrids and carried virulence genes STEC–ETEC, which are largely restricted to five serotypes (O76:H19, O43:H2, O87:H16, OR: H2, and O152:H8). Several other studies reported STEC–ETEC hybrid strains; however, unlike our study, the serotypes were O2:H27, O101:H−, O15:H16, O74:H28, O116:H28, O128:H8, O136:H12, O141:H8, O168:H8, OX182:H16, OX182:H− in Finland (23), O139, O149, O116, and OSB9 in Japan (24), O100:H− in United States (21), and O2:H2 in Burkina Faso (42).

### Antibiotic Resistance Profile

Resistance to antimicrobial agents can be a useful epidemiological marker for ETEC, STEC, and STEC–ETEC hybrid strains. In this study, the highest antimicrobial resistance was found against erythromycin 87% (20/23) followed by azithromycin 43% (10/23). In the present study, a significant proportion of ETEC and STEC strains were MDR with the highest MDR [50% (4/8)] being observed in STEC–ETEC hybrid strains. Since 1950s, antibiotics have been used in animal feed to ensure healthy livestock, prevent infections, and excel growth. Such indiscriminate use of antibiotics resulted in the widespread antibiotic resistance in bacteria (21). Erythromycin resistance during the 1990s compelled clinicians to switch to azithromycin as the drug of choice in the treatment of cholera (45) and watery (cholera-like) diarrhea caused by ETEC (46, 47).

### PFGE Analysis

As shown earlier by Vu-Khac et al. (48), a high degree of genetic polymorphisms was observed from the PFGE profiles of the STEC non-O157 and ETEC strains in the present study. Similarly, a high degree of heterogeneity was also observed with the STEC–ETEC hybrid strains, as the pulsotypes were different for strains belonging to the same serotypes, suggesting that they were unlikely to be of a single ancestral origin. A few strains showing identical PFGE patterns, however, suggest close clonal relatedness.

## Conclusion

This study is the first to show livestock as the reservoir for multidrug resistant hybrid type *E. coli* strains carrying both *stx* and *st* found in Shiga toxin-producing *E. coli* (STEC) and ETEC, respectively, in Bangladesh. Serotyping results revealed that none of the *E. coli* STEC–ETEC hybrid strains were O157:H7 but belonged to serotypes O76:H19, O43:H2, O87:H16, OR:H2, and O152:H8 and were heterogeneous genetically as confirmed by PFGE of XbaI-digested genomic DNA and cluster analyses by dendrogram. Although there may be some sorts of immunity toward Shiga toxin-producing *E. coli* existing among the healthy human population of Bangladesh (49), as they live in close proximity to the domesticated animals, multidrug resistant STEC–ETEC hybrid *E. coli* strains occurring in domesticated animals deserve careful attention as they can pose even greater public health threat for the people of Bangladesh.

## Author Contributions

AK, MO, HW, and MA contributed to the design of the study, manuscript revision, and final approval of version to be published. F-TJ designed, implemented, and performed the study in the laboratory and wrote the manuscript. SC, CG, AC, SM, SA, BH, and AN contributed to revising the manuscript critically for important intellectual content. RP, AI, AS, and MR performed the study in the laboratory. All the authors read and approved the final manuscript.

## Conflict of Interest Statement

The authors declare that the research was conducted in the absence of any commercial or financial relationships that could be construed as a potential conflict of interest.
